# Hospital doctors in Ireland and the struggle for work–life balance

**DOI:** 10.1093/eurpub/ckaa130

**Published:** 2020-09-19

**Authors:** Niamh Humphries, Aoife M McDermott, Jennifer Creese, Anne Matthews, Edel Conway, John-Paul Byrne

**Affiliations:** c1 Research Department, Royal College of Physicians of Ireland, Dublin 2, Ireland; c2 Cardiff Business School, Cardiff University, Cardiff, UK; c3 School of Nursing, Psychotherapy and Community Health, Dublin City University, Dublin Ireland; c4 DCU Business School, Dublin City University, Dublin, Ireland

## Abstract

Ireland has a high rate of doctor emigration. Challenging working conditions and poor work–life balance, particularly in the hospital sector, are often cited as a driver. The aim of this study was to obtain insight into hospital doctors’ experiences of work and of work–life balance. In late 2019, a stratified random sample of hospital doctors participated in an anonymous online survey, distributed via the national Medical Register (overall response rate 20%; *n* = 1070). This article presents a qualitative analysis of free-text questions relating to working conditions (*n* = 469) and work–life balance (*n* = 314). Results show that respondent hospital doctors, at all levels of seniority, were struggling to achieve balance between work and life, with work–life imbalance and work overload being the key issues arising. Work–life imbalance has become normalized within Irish hospital medicine. Drawing on insights from respondent hospital doctors, this study reflects on the sustainability of this way of working for the individual doctors, the medical workforce and the Irish health system. If health workforce planning is about getting the right staff with the right skills in the right place at the right time to deliver care, work–life balance is about maintaining doctor wellbeing and encouraging their retention.

## Introduction

Ireland has a high rate of doctor emigration with difficult working conditions in the Irish health system frequently cited as a key driver of doctor emigration.^1–3^ Austerity measures introduced following the 2008 global financial crisis reduced health spending,^1^ staffing levels, hospital bed numbers^4^ and new entrant salaries. One study of Irish-trained doctors who emigrated to Australia between 2008 and 2018 found that they did so in response to deteriorating working conditions^5^ in the Irish health system. Moving to Australia, which had escaped the worst effects of the global financial crisis,^1^ enabled these doctors to access better working conditions and a better work–life balance.^5^

Ireland’s medical workforce crisis is not caused by an absolute shortage of doctors, but a shortage of doctors willing to work for the terms and conditions on offer.^6^^,^^7^ Generational issues are also at play.^7^ Research with early career doctors in Ireland (2015) highlighted their dissatisfaction with the heavy, intense workloads in hospital medicine and their desire for work–life balance.^7^ It also suggests that expectations of this cohort are in line with their peers in other countries^8^ and in other professions. Employees today, ‘want work that doesn’t require substantial recovery in the evening after work, on weekends or vacations’.^9^

Workers in all occupations face competing demands on their time from work, family, friends and leisure.^10^ The literature suggests that when one aspect of life demands far more time, commitment and energy than others, it can be considered a ‘greedy institution’.^11^^,^^12^ When the workplace is a ‘greedy institution’, it can make absolute, unlimited claims ‘until no room is left in the worker’s lives for anything else’.^10^ Perhaps medicine could be considered a ‘greedy institution’^11^, first, because of the considerable demands that the hospital workplace exerts on the individual doctor (in terms of working hours, staffing levels, rotas, work intensity etc.), and second, because of the values commonly associated with the profession, i.e. an all-encompassing work identity^12^, sacrifice^13^ and workaholism.^8^

The desire for a healthy work–life balance is frequently at odds with the organization of hospital medicine. Work–life balance poses a challenge to health employers who must change the way work is organized if they are to reconcile health worker wellbeing with health service delivery.^14^ It also challenges professional cultures which have normalized work–life imbalance and poor self-care.^15^ In any industry, failing to respond to the changing employee work preferences can dent the attractiveness of ‘top jobs’^16^ and result in recruitment and retention challenges. Medicine is no different. In Ireland, the failure to address changing work preferences and improve working conditions has driven a decade of high doctor emigration.^1^^,^^17^

Drawing on free-text responses from hospital doctors surveyed in late 2019, this article aims to explore the extent to which work–life imbalance is normalized within Irish hospital medicine and to consider the impact on individual doctors, the medical workforce and the Irish health system.

## Methods

Research ethics approval was granted by the lead authors’ host institution and the survey was conducted during October–November 2019. Hospital doctors were contacted via the national Medical Register (assisted by the Medical Council of Ireland). Email invitations were distributed to 5356 hospital doctors (doctors were stratified by registration status and invitations were randomly distributed within each stratified group), inviting them to complete an anonymous online survey via Qualtrics. An overall response rate of 20% was achieved (*n* = 1070) comparable with national and international surveys of hospital doctors.^18–20^ Eighty respondents were screened out following data clean-up. Survey respondents comprised of 482 males and 479 females, which is a slight over-representation of females relative to the overall medical workforce in Ireland (42% of Ireland’s registered doctors are female).^3^ Most survey respondents (90%) reported on their experiences of working in the public sector. The survey may over-represent public sector hospital doctors. Two-thirds of Ireland’s hospital doctors work in the public sector,^21^ although dual practice (receiving a salary for treating public patients and working on a fee-basis to treat private patients), is commonplace.^21^ This article presents qualitative data generated from two free-text survey questions which previous studies have shown can yield valuable insights^2^^,^^22^ into work–life balance and working conditions. The questions were:

- Do you have any other comments on your working conditions as a hospital doctor? (*n* = 469)
- Do you have any other comments about your work–life balance? (*n* = 314)

The data generated by these two questions were pooled and imported into MaxQDA for analysis. Open coding was conducted by the lead author. The key issues raised were the impact of imbalance, long working hours and work overload. Verbatim quotes from respondents are included and respondents are referred to by survey number, followed by their gender and hospital grade, i.e. consultant or Non-Consultant Hospital Doctor (NCHD; the term is used in the Irish health system for hospital doctors who have completed their basic medical training but have not reached the grade of consultant. It includes the grades of intern, senior house officer, registrar and specialist registrar; see table 1).

**Table 1:** Profile of survey respondents

			Total
Gender	Male	482	
	Female	479	
	Other	15	976
Citizenship	Irish	707	
	Other EU	76	
	Non-EU	193	976
Current grade	Consultant	289	
	NCHD	680	969
Caring responsibilities	Yes	430	
	No	546	976
Public/private hospital	Public hospital	864	
	Private hospital	32	
	Both	65	961

## Results

At all career stages, survey respondents (both male and female) were struggling to achieve a work–life balance. Seventy-three percent of survey respondents agreed or strongly agreed with the statement ‘I often feel the strain of attempting to balance my responsibilities at home and at work’, and this was slightly higher (78%) for female respondents. Of the free-text responses received, most were negative. This is consistent with the work–family conflict measures also included in the survey, which found high work–life conflict scores across all demographics and hospital grades.^23^

### Impact of imbalance on own wellbeing

Respondent hospital doctors struggled to achieve a work–life balance. They felt that long and unpredictable working hours, combined with a high level of work intensity, left them with no time for anything else, as this respondent explains:

‘No work-life balance. Routinely work beyond rostered hours … worn out by the end of the working day, no energy or motivation for anything except to eat and go to bed. No real interest in doing anything as time feels better spent recouping before going back to work. Have isolated self from friends/missed family events through work/call commitments’ (Respondent 354/Senior House Officer/M).

Respondents articulately described the impact of work–life imbalance on their families. They were frustrated at the need to prioritize work at the expense of everything else. ‘While I don’t mind working long hours, it is frustrating that I can never make outside commitments such as sport or leisure’ (Respondent 165/Intern/M). ‘I have no life. I’ve lost a lot of friends. It is my work’ (Respondent 206/Senior House Officer/M).

Senior hospital doctors also struggled to achieve balance, indicating that work–life imbalance is not exclusively an early career issue. Respondents associated their heavy, intense workloads with their inability to disconnect from work. ‘I feel if we were under less pressure during the day, we would operate better and be more able to switch off at home’ (Respondent 515/Consultant/F). Respondents who mentioned balance felt that, first, they did not have a work–life balance and, second, that work–life balance was not prioritized in Ireland’s hospitals; ‘work-life balance is neither promoted nor encouraged by the hospitals, or those working in them’ (Respondent 606/Specialist Registrar/F).

### Impact of imbalance on others

Early career hospital doctors adapted quickly to this way of working by prioritizing work and sacrificing time with their partners, family and friends.

‘I … abstain completely from non-work activities for weeks at a time. I go to work, I go home, I sleep, I go to work … and I see my wife every other week’ (Respondent 230/Senior House Officer/M).

When work impeded their ability to manage family and caring commitments, frustration morphed into distress and respondents (both male and female), described the struggle to manage family responsibilities alongside professional commitments. ‘As a full-time consultant, wife, mother and daughter of elderly parent there is no time left for me because of all the competing demands’ (Respondent 340/Consultant/F).

‘I feel tired and burnt out by the time I get home to see my wife and children. I don’t feel that I am being a good father at the moment’ (Respondent 831/Specialist Registrar/M).

Forty-four percent of survey respondents had caring responsibilities outside of work (see table 1). Work also impacted on respondents’ capacity for self-care.

‘I am so exhausted I just come home and sleep, I don’t have time to cook properly or exercise: I find I cannot justify taking time off work to attend routine GP appointments and I am often in work so late and on the weekends that I sometimes can’t even get my prescriptions for my antidepressants filled’ (Respondent 551/Senior House Officer/F).

‘It is unsafe to work for 24 hours straight. It’s not safe for patients or for us. I have nearly fallen asleep at the wheel multiple times on the way home post-call’ (Respondent 325/Registrar/F).

In contrast to the post-call exhaustion described above, respondents also explained how work-related anxiety affected their ability to relax, or to sleep. ‘Being upset about how this job has evolved affects my sleep, confidence, and stress levels’ (Respondent 496/Consultant/F). Respondents explained, in stark terms, the negative impact that work–life imbalance has on their lives, even for those at the very start of their medical careers.

‘I don’t have a work-life balance … I cry myself to sleep at least once a week because of the frustration and anger I feel at my job and because of the impact it has on every other aspect of my existence’ (Respondent 68/Specialist Registrar/F).

‘I feel anxious all the time in this post … I have a constant central chest pain when I’m at work and sometimes at home … I have nightmares most nights (5/7 nights) about work related themes. This is unsustainable. I am an intern and feel burnt out’ (Respondent 552/Intern/F).

### Seeking better balance

Although a minority of respondents articulated aspects of the work that they enjoyed, few were positive about their work–life balance.

‘I love my job, I really do but it absolutely dominates my life’ (Respondent 709/Senior House Officer/M).

‘I do love my job, I love medicine and thoroughly enjoy being in the hospital and learning. I don’t want to have to choose between my career and functioning as a normal human’ (Respondent 386/Senior House Officer/F).

Respondents considered their options for achieving work–life balance. The solutions under consideration included emigration and retirement. ‘I’m exhausted. It took me a year to realize that I need out’ (Respondent 206/Senior House Officer/M). ‘I really don’t know if I will stay in hospital medicine. I like the work, but they make the job too hard’ (Respondent 544/Registrar/F). Hospital doctors, at the start and nearing the end of their careers, were concerned about the impact of work–life imbalance on their future.

‘I feel my future of working in the HSE looks very bleak and I can’t fathom the idea of trying to have a normal family life alongside a job in hospital medicine in Ireland’ (Respondent 14/Senior House Officer/F).

‘It is not a sustainable imbalance for a full career … We should be able to navigate with respect through an alternative fourth quarter career rather than early retirement’ (Respondent 209/Consultant/F).

Despite their dissatisfaction with the status quo, respondents expressed little hope for improvement. ‘It’s easier to get on with it than put time and effort into changing things that are not in my power to change’ (Respondent 391/Specialist Registrar/M). ‘I have never seen conditions as bad and am concerned we have all begun to expect and accept them’ (Respondent 48/Consultant/M). These quotes imply that respondents are resigned to work–life imbalance and/or that they lack the time or energy needed to initiate change.

## Discussion

The overwhelming sense from the data is that work–life imbalance is standard in Irish hospital medicine. This is in line with the quantitative survey findings^23^ and also with previous research internationally^19^ and in Ireland—where only one in five hospital doctors surveyed were satisfied with their work–life balance.^24^ This article illustrates the distress caused by work–life imbalance and its impact on male and female respondents. Although the work–life balance debate often focuses on the experiences of mothers of young children, our results highlight that work–life imbalance is a problem for both men and women and those with any caring responsibilities.^25^ The question is why such unsustainable ways of working appear so commonplace in Irish hospital medicine, and what the implications are for the individual doctors, for the medical workforce and for the health system?

The debate continues as to whether medicine is a vocation,^26^ a calling^27^ or a highly skilled job.^26^ However, when medicine-as-vocation is used to justify working conditions deemed unacceptable in any other profession,^27^ it becomes problematic. Reflecting on hospital medicine in the United Kingdom, for instance, Kay has asked whether there are other professions that require workers to arrange their own sickness cover^28^ and describes hospitals as reliant upon ‘the charity of doctors staying beyond their contracted hours to get things done’.^28^

That respondents consider work–life balance unachievable and frequently put work ahead of their own wellbeing, is a cause for concern and reminiscent of the ‘greedy institution’^10–12^ which demands too much of its workers. Qualitative research from the USA outlines how work–life balance in a medical context was taken to mean that life outside work should be managed so as not to impede work and notes how medics were ‘groomed’ for a life of work–life conflict.^12^ Perhaps Ireland’s hospital doctors are socialized to expect work–life imbalance, an expectation then reinforced organizationally by staffing levels and hospital rotas. Writing in the *British Medical Journal*, Kar^26^ suggests that the medical profession should remind the public that being a doctor is a tough job, but one from which doctors need the occasional break. Perhaps hospital medicine needs to replace the greediness, with policies and cultures^10^ more supportive of hospital doctors and their right to a work–life balance.

International research has found that conflict between the personal and the professional inform decisions to reduce work hours and even to exit medicine.^19^ Ireland has a high rate of doctor emigration.^1^ Over the past decade many doctors, dissatisfied with their working conditions, emigrated.^1^^,^^7^ Perhaps their emigration released pent-up dissatisfaction from the system and enabled work–life imbalance as standard to continue as doctors opted for emigration rather than voice.^29^ In response to exit by emigration, Ireland increased its reliance on internationally trained doctors. Accounting for 43% of the medical workforce,^3^ internationally trained doctors have enabled Ireland to change the doctors in the system instead of changing the system^30^ by improving hospital doctors’ working conditions.

### Limitations

The survey findings represent the views of a subset of Irish hospital doctors (*n* = 1070) at a specific point in time (October/November 2019). There is a risk that hospital doctors with more negative experiences were more likely to respond to the survey. Although the response rate is low (20%), it is keeping with previously published studies of hospital doctors in Ireland and internationally.^18–20^ The survey had a low response from hospital doctors working solely in private hospitals.

## Conclusion

Our study highlights the importance of work–life balance to support hospital doctor wellbeing and encourage retention. Most survey respondents (73%) were feeling the strain of work–life imbalance and respondents clearly articulated the negative impact of this on their lives and wellbeing. Although dissatisfied with their work–life imbalance, respondents accepted it as the norm within hospital medicine. This survey was conducted in late 2019. The 2019 novel coronavirus (COVID-19) pandemic puts the findings into sharp relief. It is clear that hospital doctors at the COVID-frontline will need decompression time and adequate time off-work to care for themselves and their families, as well as their patients during the weeks and months ahead.^31^ Facilitating self-care and work–life balance for hospital doctors may prove to be as critical to the COVID-19 response, as protocols and protective equipment.^31^ In this regard, hospital organizations must become more adept at listening, and responding, to the needs of the medical workforce. If health workforce planning is about getting the right staff with the right skills in the right place at the right time to deliver care,^32^ work–life balance is about maintaining their wellbeing and encouraging their retention. Work–life balance must be factored into medical workforce models and hospital rotas if Ireland is to retain a motivated medical workforce into the future.

**Key points**

- Ireland has a high rate of doctor emigration, with doctors emigrating to achieve better working conditions internationally.
- Work–life imbalance has become normalized in hospital medicine in Ireland.
- Respondents, both male and female, explained how work–life balance was impacting on their wellbeing and on their relationships.
- Work–life balance must be factored into medical workforce models and hospital rotas to retain a motivated medical workforce.
